# Examining plasma microRNA markers for colorectal cancer at different stages

**DOI:** 10.18632/oncotarget.7196

**Published:** 2016-02-04

**Authors:** Yan Sun, Yuexin Liu, David Cogdell, George A. Calin, Baocun Sun, Scott Kopetz, Stanley R. Hamilton, Wei Zhang

**Affiliations:** ^1^ Department of Pathology, The University of Texas MD Anderson Cancer Center, Houston, TX 77030, USA; ^2^ Department of Experimental Therapeutics, The University of Texas MD Anderson Cancer Center, Houston, TX 77030, USA; ^3^ Department of Gastrointestinal Medical Oncology, The University of Texas MD Anderson Cancer Center, Houston, TX 77030, USA; ^4^ The Center for RNA Interference and Non-Coding RNAs, The University of Texas MD Anderson Cancer Center, Houston, TX 77030, USA; ^5^ Department of Pathology, Tianjin Medical University Cancer Institute and Hospital, Tianjin 300060, China

**Keywords:** microRNA, biomarker, plasma, colorectal cancer, stage

## Abstract

Circulating microRNAs (miRNAs) have emerged as promising biomarkers; however, few miRNAs have been reproducible and can be used in clinical practice. In this study, we screened the levels of 754 miRNAs using TaqMan array in 50 individual plasma samples from 10 demographically matched healthy controls and 40 colorectal cancer (CRC) patients (10 each of stage I–IV) and identified 22 miRNAs associated with the presence of and stages of CRC. Then we performed the validation for 11 miRNAs in an independent cohort including 187 CRC cases and 47 healthy controls. Comprehensive analyses showed that plasma miR-96 distinguished stage I–IV CRC from healthy controls with an area under curve (AUC) of 0.740; miR-203 separated stage III–IV CRC patients from stage I–II with an AUC of 0.757; and miR-141 differentiated stage IV CRC from stage I–III patients with an AUC of 0.851. Survival analyses showed that plasma miR-96 and miR-200b were independent prognostic factors for overall survival. Thus, we propose four miRNAs (miR-96, miR-203, miR-141 and miR-200b) as clinically validated circulating biomarkers for CRC prognosis that warrant further evaluation for clinical utility.

## INTRODUCTION

Colorectal cancer (CRC) remains the third most commonly diagnosed cancer in both men and women and it is the third leading cause of cancer death in the United States: an estimated 136,830 people were diagnosed and 50,310 people died of the disease in 2014 [1]. The main challenges in reducing the mortality rate are that CRC is asymptomatic in the early stages, there is no effective method of monitoring recurrence after treatment for early-stage CRC, and treatment for recurrent and metastatic CRC is suboptimal. Colonoscopy screening has contributed to the early detection of CRC and a decrease in mortality in recent years [2]. However, the invasive nature and relatively high cost of the procedure have hampered its application globally. Fecal occult blood test, although less invasive, has lower sensitivity (23.9%) [3]. Screening with carcinoembryonic antigen (CEA) levels in the blood also has poor sensitivity (36–74%, based on CRC stage) [4]. Therefore, the identification and validation of non-invasive circulating markers for CRC detection, monitoring, and prognosis remain an incomplete aspect of CRC research.

MicroRNAs (miRNAs) are small, non-coding RNAs that regulate the expression of target genes through an RNA-interfering mechanism or translational inhibition [5]. It is well established that aberrant expression of miRNAs is associated with cancer development, progression and treatment [6, 7]. A number of studies have identified some miRNAs as potential circulating biomarkers for the diagnosis and prediction of CRC (Table S1) [8–23]. However, few miRNAs have been reproducible among studies. This discordance may be because most studies had a limited sample size, did not include CRC patients with all major clinical stages of disease, evaluated only a limited number of candidate miRNAs, and used different assay methodologies and normalizers.

**Figure 1:**
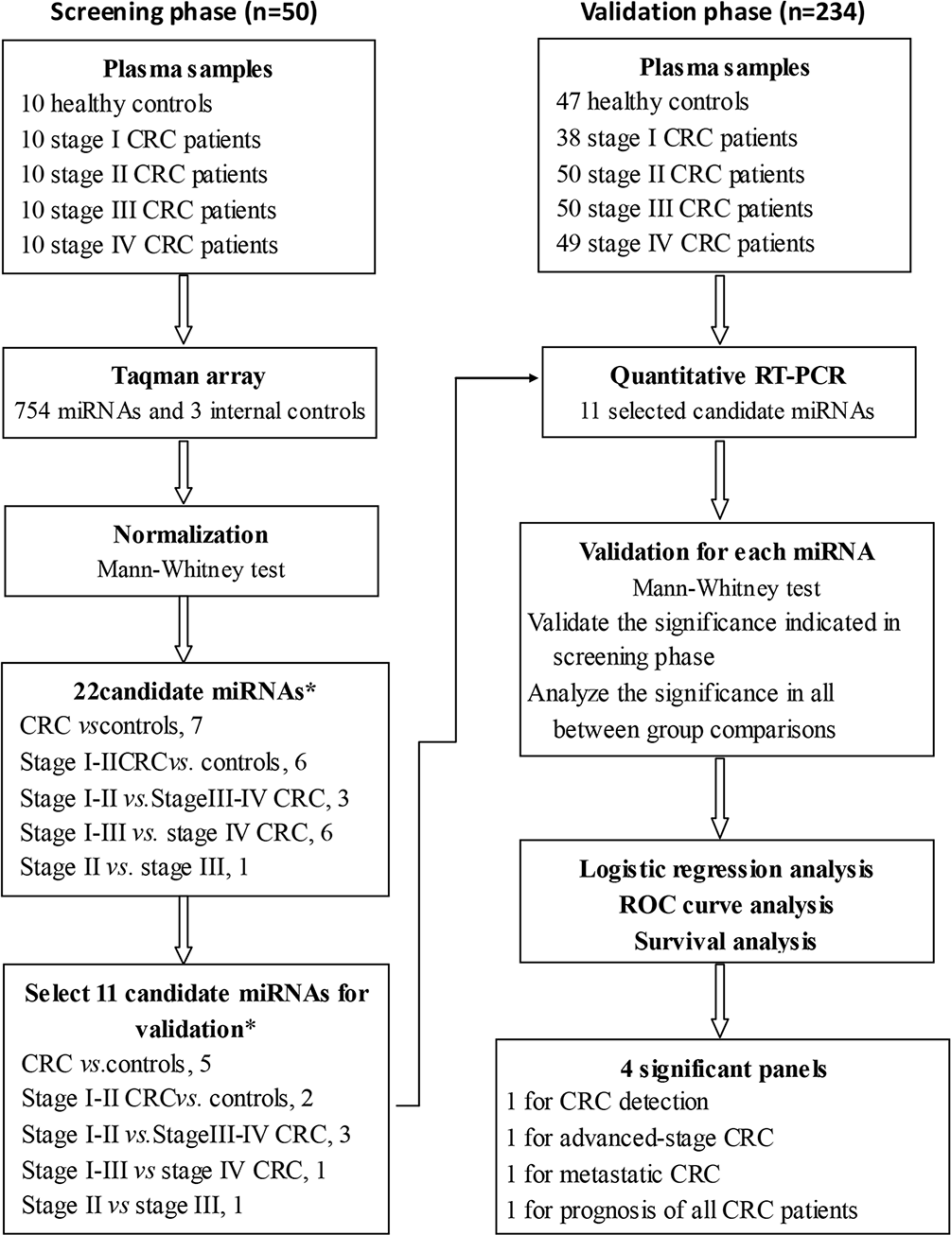
Study design The microRNA (miRNA) profiles of 284 plasma samples from 227 CRC patients and 57 healthy controls were used to generate outcomes in 2 different phases. The candidate miRNAs discovered on 50 plasma samples using Taqman arrays were validated in 234 plasma samples using quantitative RT-PCR. The logistic regression, ROC curve and survival analyses were performed in the validation cohort. CRC: colorectal cancer. *1 miRNAs repeated to be candidates in different comparisons.

To overcome the limitations of existing studies of circulating miRNAs in patients with CRC, we performed a two-step discovery and clinical validation study with a comprehensive statistical analysis in a large number of patients with all stages of CRC and in healthy controls. The design of this study is shown in the flow diagram in Figure 1. In the first step, we measured the levels of 754 miRNAs in 50 plasma samples, including 10 healthy controls and 10 stage I, 10 stage II, 10 stage III, and 10 stage IV CRC patients. On the basis of an analysis to select suitable internal reference miRNAs, we evaluated plasma miRNAs that had potential for the detection of the presence of CRC and for associations with clinical outcomes based upon stage of disease. We identified 22 miRNAs that were most differentially expressed in the plasma of healthy controls and all CRC patients or early stage (stage I–II) patients; in early stage and late stage (stage III–IV) patients; in stage I–III and patients with metastatic disease (stage IV); and in stage II and III CRC patients for which prognosis is important in decisions regarding post-operative adjuvant chemotherapy. In the clinical validation step, 11 miRNAs were examined in 234 plasma samples from CRC patients with follow-up data and from healthy controls. A comprehensive analysis revealed plasma miRNAs that may be useful for CRC prognosis after establishment of clinical utility.

## RESULTS

### Study population

The discovery set consisted of 40 CRC patients (10 each of stage I–IV) and 10 healthy controls. All cases were age- and sex-matched, and no significant differences were seen in clinical parameters between the selected cases and the remaining patients in each comparison group (Table 1). Two hundred thirty-four cases, including 47 healthy controls and 187 CRC patients, were used to validate selected miRNAs. Their characters were shown in Table 1.

**Table 1:** Characteristics of healthy controls and colorectal cancer patients in this study

Variable	Screening cohort	Validation cohort	*P* value
**Healthy control, *n***	10	47	
**Sex, *n* (%)**			0.854
Male	5 (50)	22 (46.8)	
Female	5 (50)	25 (53.2)	
**Age (mean ± SD)**	54 ± 6.4	54 ± 6.3	0.913
**CRC patients, *n***	40	187	
**Sex, *n* (%)**			0.735
Male	20 (50)	99 (52.9)	
Female	20 (50)	88 (47.1)	
**Age (mean ± SD)**	55 ± 6.3	55 ± 7.8	0.773
**Stage, *n* (%)**			0.933
I	10 (25)	38 (20.3)	
II	10 (25)	50 (26.7)	
III	10 (25)	50 (26.7)	
IV	10 (25)	49 (26.2)	
**Surgery, *n* (%)**			0.887
Yes	33 (82.5)	156 (83.4)	
No	7 (17.5)	31 (16.6)	
Chemotherapy*, *n* (%)			0.898
Yes	31 (77.5)	144 (77.0)	
No	9 (22.5)	42 (22.5)	
**Status, *n* (%)**			0.420
Living	31 (77.5)	128 (68.4)	
Dead	8 (20.0)	46 (24.6)	
No information	1 (2.5)	13 (7.0)	

* No chemotherapy information was available for one patient.

### Identification of candidate miRNAs in discovery study

Using the TaqMan^®^ Array Human MicroRNA A + B Cards Set v3.0, we profiled the levels of 754 miRNAs in 50 plasma samples from 10 healthy controls and 40 CRC patients (10 each of stage I–IV). The raw data and processed data were summarized in Table S2. In the analysis, we first determined the normalization reference, because no standard reference miRNA has been established for the studies of circulating miRNAs [24]. Although the TaqMan array provides U6, RNU44, and RNU48 as internal controls, which are often used in the normalization for cellular miRNAs, the levels of RNU44 and RNU48 were too low across all samples to be useful (Figure S1A). We therefore calculated the standard deviations of expression of all the miRNAs and found that six in card A (miR16, miR-17, miR-103, miR-192, miR-451, and miR-93) and six in card B (miR-877, miR-188, miR-138-1, miR-520c-3p, miR-610, and U6) had relatively high RNA yields and the least expression variation across the discovery cohort (Figure S1B and S1C). Further analyses showed that the expression levels of miR-451 and miR-877 were not significantly different between healthy controls and CRC patients (Table S3) and were not significantly associated with tumor stage, and were therefore chosen as the normalization references to quantify plasma miRNAs in cards A and B, respectively.

**Figure 2:**
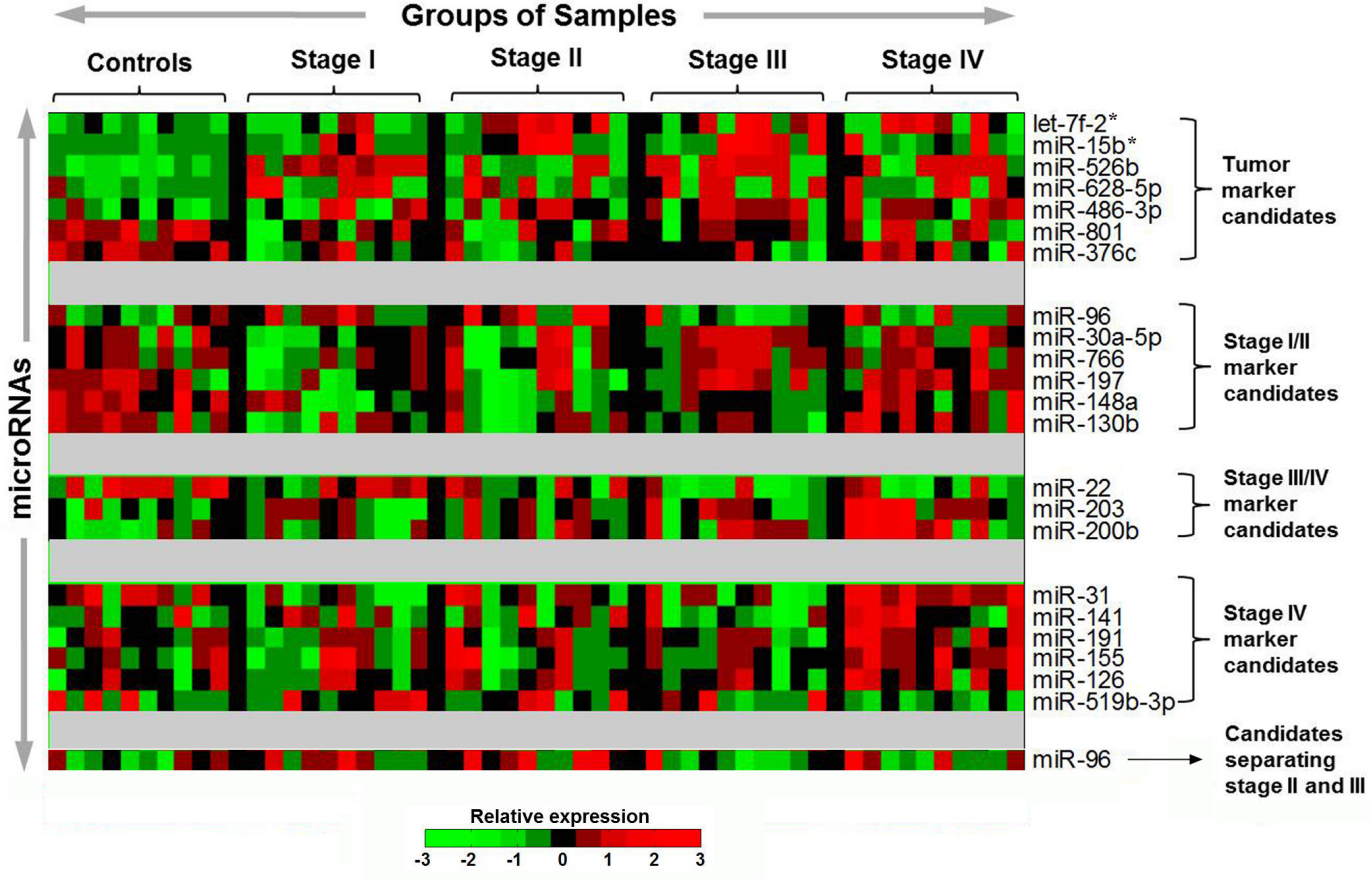
Potential plasma microRNA candidates selected from the TaqMan microarrays with 754 human miRNAs in the discovery study The heatmap shows the relative expression pattern of the potential miRNA marker candidates across the 10 healthy controls and 40 CRC patients. The patient samples were grouped and ordered on the basis of tumor stage (10 samples per each of stage I–IV). Each row corresponds to plasma miRNA and each column corresponds to an individual sample. Expression levels of each miRNA are normalized across the samples such that the mean is zero and the standard deviation is equal to 1. Expression levels greater than the mean are shaded in red, and those below the mean are shaded in green. The miRNAs on the row were grouped on the basis of different biomarker categories.

We performed several pairwise comparisons in the individual 50 samples and identified 22 miRNAs with differential expression levels between groups (Figure 2, Table S4). Among the seven miRNAs that exhibited different expression levels between CRC patients and controls, let-7f-2* (*P* = 0.008), miR-15b* (*P* < 0.001), miR-526b (*P* < 0.001), miR-628-5p (*P* < 0.001), and miR-486-3p (*P* < 0.001) had higher levels in CRC patients, respectively, while miR-801 (*P* < 0.001) and miR-376c (*P* < 0.001) had higher levels in controls. Six miRNAs had distinctive expression in early-stage CRC, including higher level of miR-96 (*P* = 0.031) and lower levels of miR-30a-5p (*P* = 0.037), miR-766 (*P* = 0.027), miR-197 (*P* = 0.039), miR-148a (*P* < 0.001), and miR-130b (*P* = 0.002), respectively. In advanced stage CRC, miR-203 (*P* = 0.035) and miR-200b (*P* = 0.005) were relatively enriched, whereas miR-22 (*P* = 0.003) was lower. Five miRNAs, including miR-31 (*P* < 0.001), miR-191 (*P* = 0.006), miR-155 (*P* < 0.002), miR-126 (*P* = 0.023) and miR-141 (*P* = 0.056), were markedly higher or showed a trend of being higher in stage IV CRC, while miR-519b-3p had lower levels (*P* = 0.003) in stage IV CRC. In addition, we found that miR-96 levels were higher in stage II than in stage III (*P* = 0.024).

### Clinical validation of candidate miRNAs in a second large cohort of CRC patients and controls

We selected the 11 miRNAs for clinical validation in a larger cohort (187 CRC cases and 47 healthy controls) using quantitative RT-PCR and spiked-in cel-miR-39 as a normalizer. We first validated miR-141 since it was indicated to be a biomarker for metastatic CRC in our previous study [11]. Consistently, plasma miR-141 levels were higher in stage IV CRC patients than in stage I–III (*P* < 0.001, Figure S2A). A further detailed analysis at each stage showed that the plasma miR-141 levels in stage IV CRC patients were significantly higher than in controls and stage I, stage II, and stage III CRC patients, respectively (*P* < 0.001 for all, Figure S2B). In addition, CRC patients of all stages had higher miR-141 levels than did controls (*P* < 0.001, Figure S2C), and miR-141 levels of stage III–IV patients were higher than those of controls and stage I–II patients (*P* < 0.001, Figure S2D). The detailed data were also seen in Table S5.

**Figure 3:**
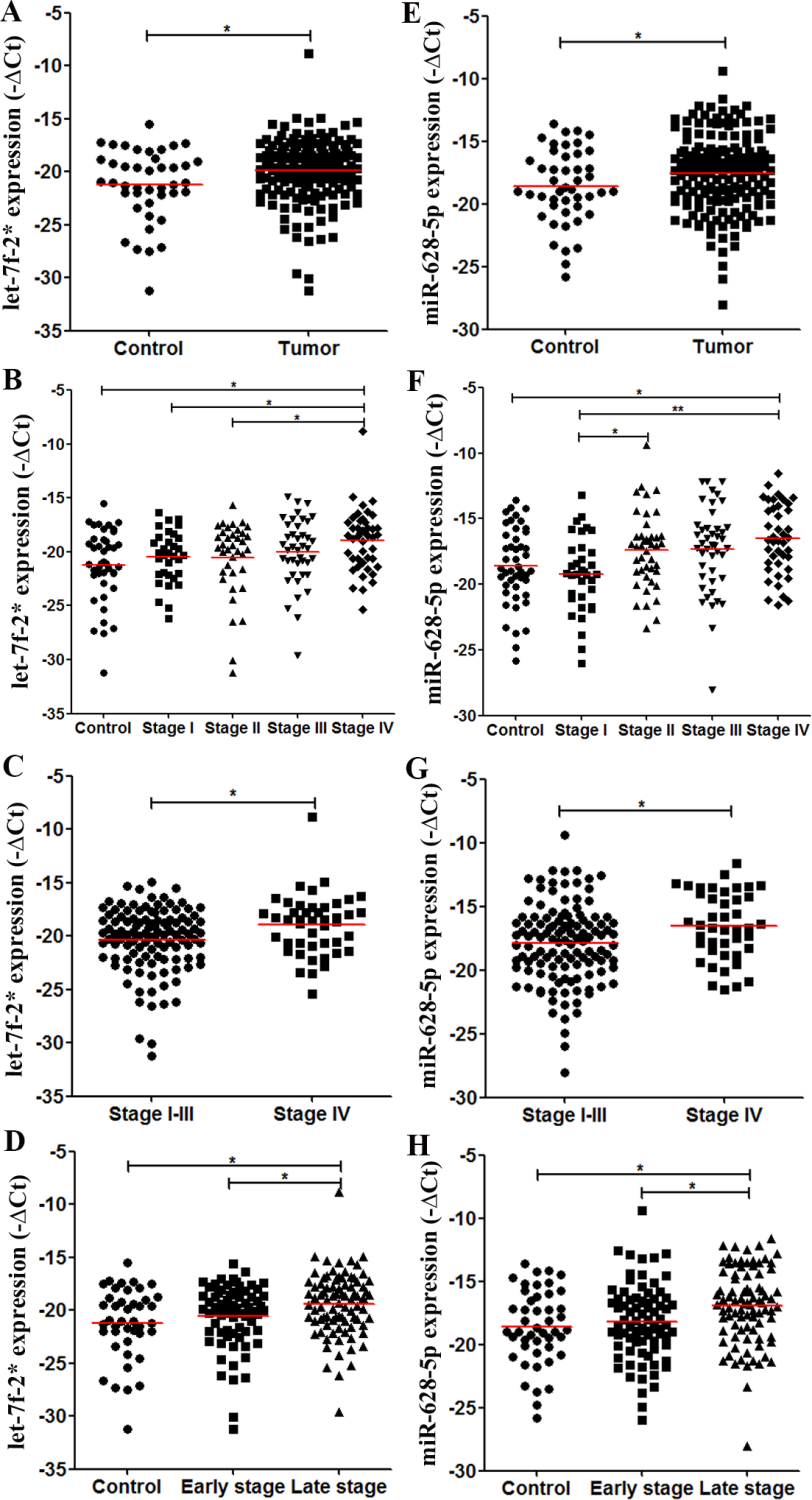
Levels of plasma miRNA candidates as potential CRC markers in the validation cohort The levels of plasma miRNAs from 187 CRC patients and 47 healthy controls were examined using real-time RT-PCR and normalized with cel-miR-39 as the control. (**A**–**D**) Levels of plasma let-7f-2* in healthy controls and all CRC patients (A); in controls and each stage of CRC patients (B); in stage I–III and stage IV CRC patients (C); and in controls, stage I–II and stage III–IV CRC patients (D). (**E**–**H**) Levels of plasma miR-628-5p in multiple comparisons same with (A–D). *P < 0.05; **P < 0.01. Exact *p*-values for each comparison were listed in Table S5.

We compared the levels of plasma let-7f-2*, miR-15b*, miR-526b, miR-628-5p, and miR486-3p between CRC patients and controls which were suggested to be different in the discovery phase, and demonstrated the significant difference of let-7f-2* (*P* = 0.019, Figure 3A) and miR-628-5p (*P* = 0.037, Figure 3E) levels in the validation cohort (Table S5). A detailed analysis further revealed that only stage IV CRC patients had significantly higher let-7f-2* (*P* = 0.001, Figure 3B) and miR-628-5p (*P* = 0.001, Figure 3F) levels than did controls (Table S5). Moreover, the CRC patients in stage IV had higher let-7f-2* and miR-628-5p levels than those with stage I–III CRC (*P* = 0.010, Figure 3C; *P* = 0.010, Figure 3G). In addition, both let-7f-2* and miR-628-5p levels were significantly higher in stage III–IV than those in stage I–II CRC patients (*P* = 0.026, Figure 3D; *P* = 0.006, Figure 3H). Although there was no significant difference in plasma miR-15b*, miR486-3p, and miR-526b levels between controls and CRC patients, miR-15b* and miR-526 levels were significantly higher in stage IV CRC patients than in controls (Table S5).

**Figure 4:**
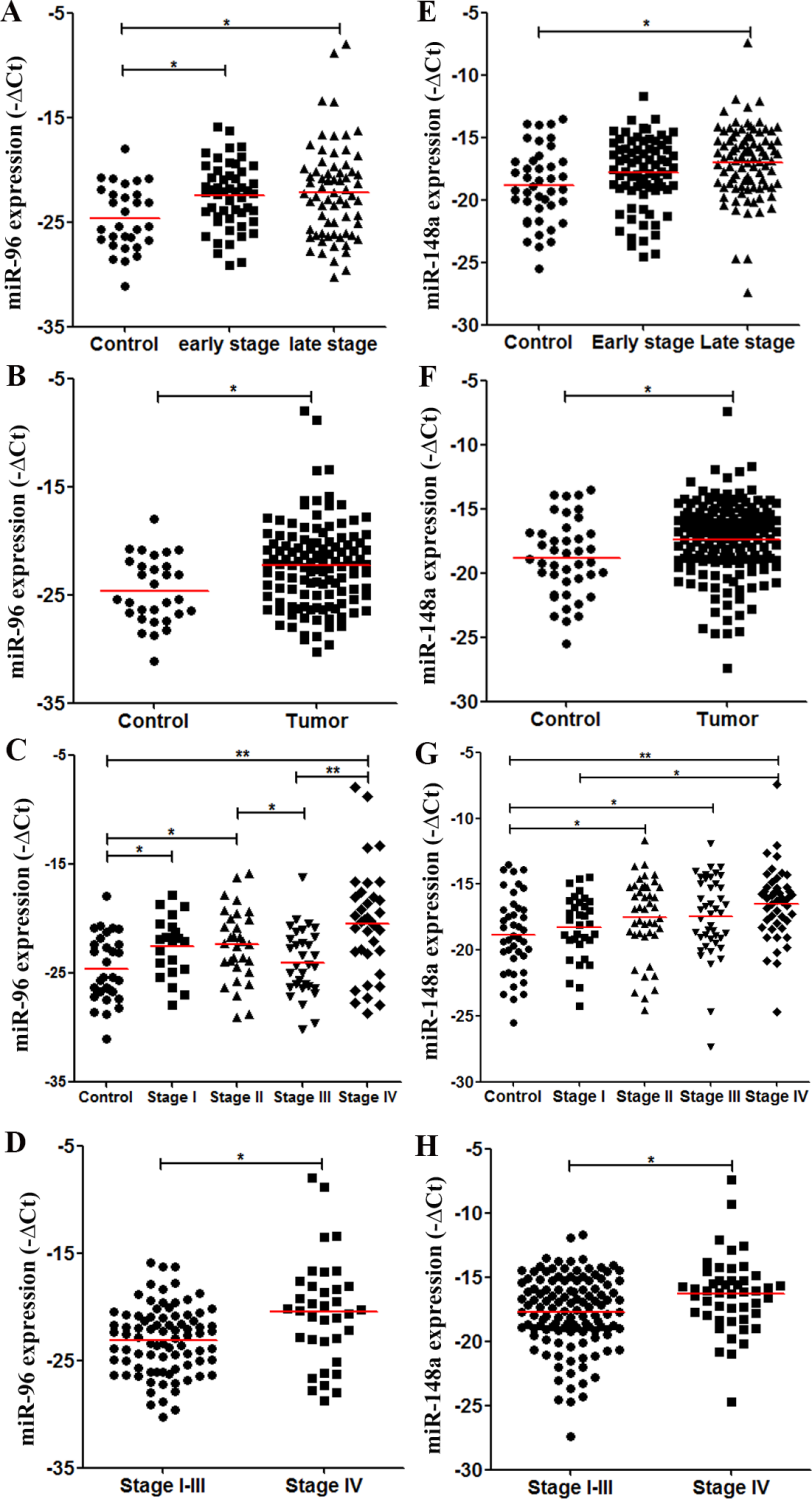
Levels of plasma miRNA candidates as potential early-stage CRC markers in the validation cohort (**A**–**D**) Levels of plasma miR-96 in healthy controls, stage I–II and stage III–IV CRC patients (A), in healthy controls and all CRC patients (B), in healthy controls and each stage CRC patients (C), and in stage I–III and stage IV CRC patients (D). (**E**–**H**) Levels of plasma miR-148a in multiple comparisons same with (A–D). *P < 0.05; **P < 0.01. Exact *p*-values for each comparison were listed in Table S5.

We performed the validation of miR-96 and miR-148a as candidates of early-stage CRC markers. Consistent with the discovery results, miR-96 levels were significantly higher in stage I–II CRC than in controls (*P* = 0.003, Figure 4A). Plasma miR-96 levels in stage III–IV CRC patients were also higher than those in controls (*P* = 0.007, Figure 4A). Therefore, all CRC patients had higher miR-96 levels than did controls (*P* = 0.003, Figure 4B). A further analysis showed that compared with controls, miR-96 levels were higher in stage I (*P* = 0.019), stage II (*P* = 0.009), and stage IV CRC patients (*P* < 0.001). Interestingly, plasma miR-96 decreased from stage II to stage III (*P* = 0.043), and then showed the highest levels in stage IV (Figure 4C). CRC patients in stage IV had higher plasma miR-96 levels than stage I–III patients (*P* = 0.001, Figure 4D). Inconsistent with the discovery data, plasma miR-148a showed the trend of having higher levels in stage I–II CRC than in controls in validation cohort (*P* = 0.064, Figure 4E). Furthermore, miR-148a levels in stage III–IV CRC patients were higher than in controls (*P* = 0.001, Figure 4E). Altogether, miR-148a levels were higher in all CRC patients than in controls (*P* = 0.003, Figure 4F). In each group, miR-148a levels were significantly higher in stage II (*P* = 0.041), stage III (*P* = 0.037), and stage IV CRC patients (*P* < 0.001) than in controls (Figure 4G). In addition, miR-148a levels were higher in stage IV patients than in stage I–III (*P* = 0.006, Figure 4H). The detailed data were also seen in Table S5.

**Figure 5:**
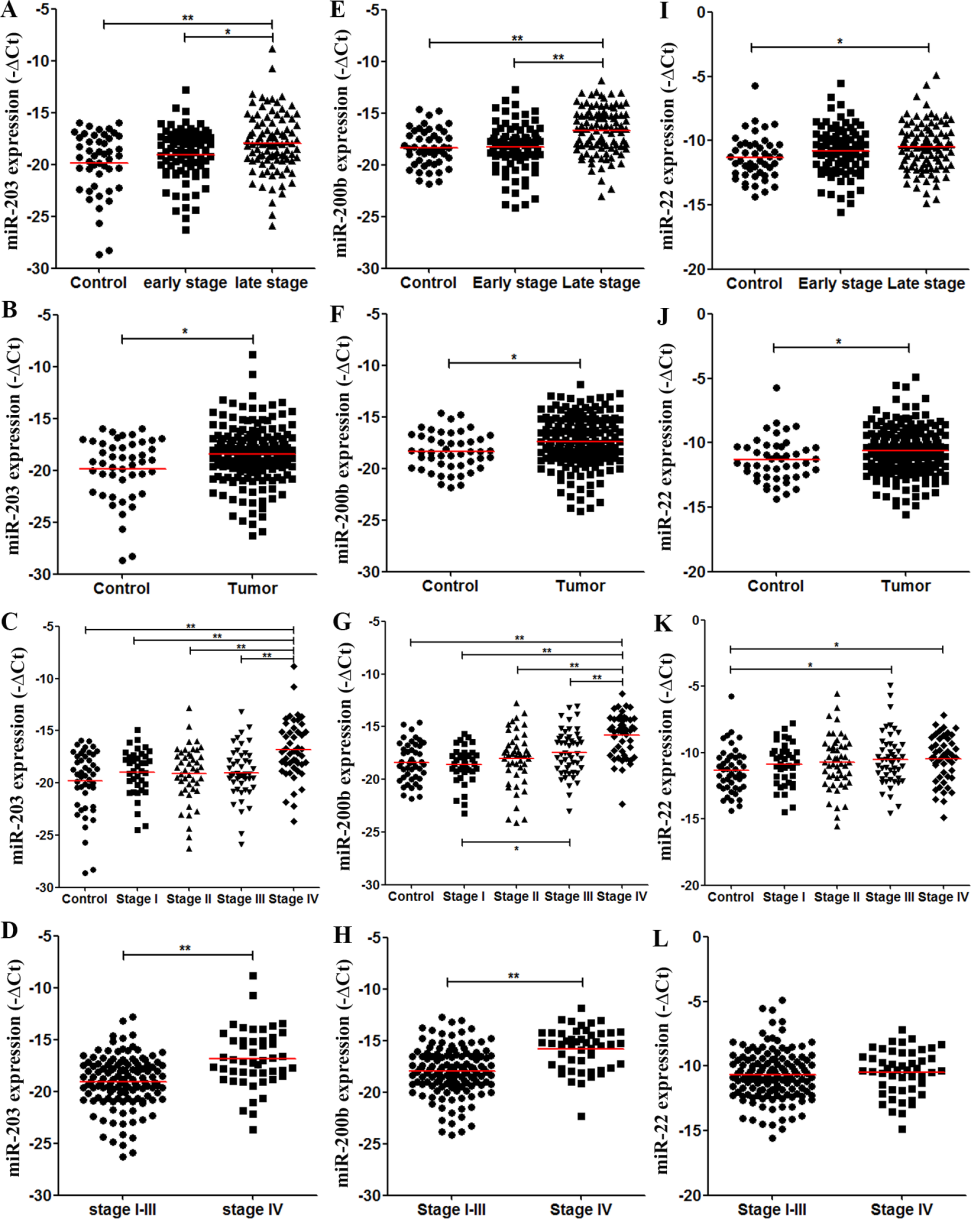
Levels of plasma miRNA candidates as potential late-stage CRC markers in the validation cohort (**A**–**D**) Levels of plasma miR-203 in healthy controls, stage I–II and stage III–IV CRC patients (A), in healthy controls and all CRC patients (B), in controls and each stage CRC patients (C), and in stage I–III stage and IV CRC patients (D). (**E**–**H**) Levels of plasma miR-200 b in multiple comparisons same with (A–D). (**I**–**L**) Levels of plasma miR-22 in multiple comparisons same with (A–D). *P < 0.05; **P < 0.01. Exact *p*-values for each comparison were listed in Table S5.

Consistent with the discovery results, plasma miR-203 and miR-200b levels were significantly higher in stage III–IV CRC patients than in controls (*P* < 0.001 for both) and stage I–II CRC patients (*P* = 0.006 and *P* < 0.001, respectively) in the validation cohort (Figure 5A, 5E). CRC patients had higher miR-203 and miR-200b levels than did controls (*P* = 0.003, Figure 5B; *P* = 0.012, Figure 5F). Plasma miR-203 and miR-200b levels were significantly higher in stage IV CRC patients, not in stage III, than in controls and each stage CRC patients (Figure 5C, 5G). Altogether, stage IV CRC patients had higher plasma miR-203 and miR-200b levels than the patients of stage I–III (*P* < 0.001 for both, Figure 5D, 5H). In contrast to the result form discovery phase, plasma miR-22 levels were higher in stage III–IV CRC patients than in controls in the validation cohort (*P* = 0.009, Figure 5I). They were also significantly higher in CRC patients than in controls (*P* = 0.019, Figure 5J). Compared with controls, both stage III and IV CRC patients had higher miR-22 levels (*P* = 0.027, *P* = 0.016, respectively; Figure 5K).

Same with the results of the discovery phase, miR-96 levels were significantly higher in stage II than stage III CRC patients in the validation cohort (*P* = 0.043, Figure 4C).

Plasma miR-451 levels were also validated in the validation cohort to determine whether it was stable across all samples. The results showed that there was no significant difference in miR-451 levels between each group (*P* > 0.05 for all, Figure S3). In addition, we performed the same analysis for the 11 miRNAs using miR-451 as an endogenous control and found that the results were similar to those using cel-miR-39 as a control (Table S6).

### Potential microRNAs for CRC detection and stage

**Figure 6:**
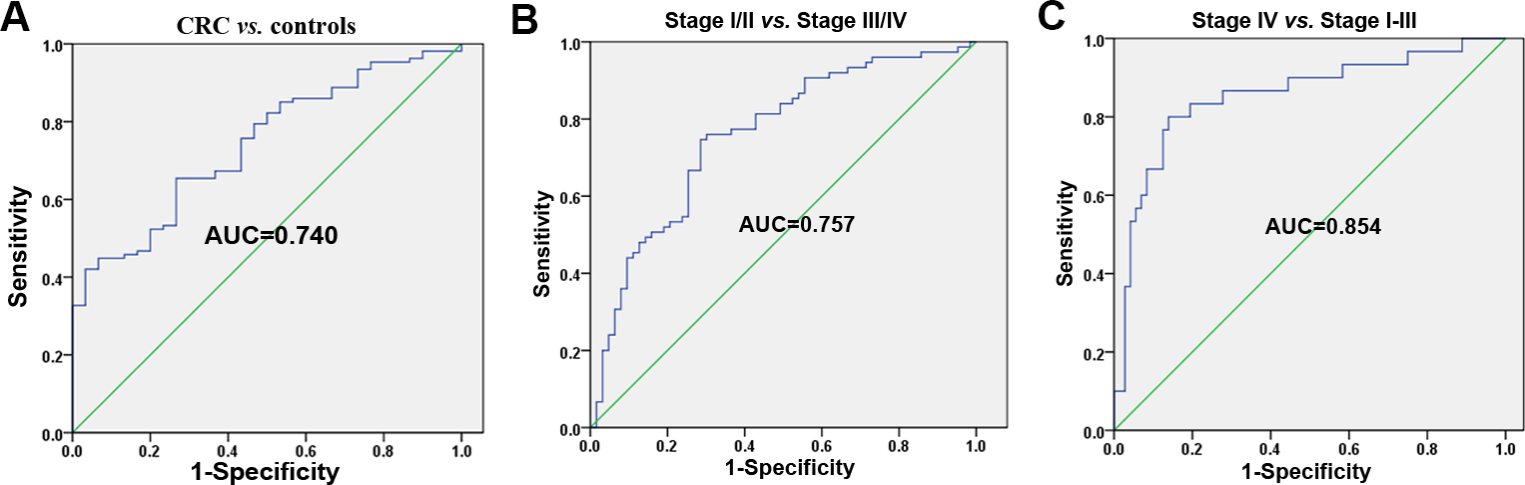
Integrated analyses of the potential microRNAs for CRC detection and stage in the validation cohort (**A**) ROC analysis of the logistic regression model based on plasma miR-96 for separating CRC patients (*n* = 187) from healthy controls (*n* = 47). (**B**) ROC analysis of the logistic regression model based on plasma miR-203 for differentiating stage III–IV (*n* = 99) from stage I–II CRC patients (*n* = 88). (**C**) ROC analysis of the logistic regression model based on plasma miR-141 for separating stage IV (*n* = 49) from stage I–III CRC patients (*n* = 138).

We performed a combined analysis with each candidate miRNA in the validation set. The above results from the validation cohort showed that plasma levels of let-7f-2*, miR-628, miR-96, miR-148a, miR-203, miR-200b, miR-22 and miR-141 were significantly different between all CRC patients and controls. A logistic regression analysis identified the best model based on miR-96, logit (p) = 12.918 + 0.149* miR-96. At the optimal cut-off, the predicated probability based on plasma miR-96 levels had 65.4% sensitivity and 73.3% specificity in distinguishing between all CRC patients and controls, with an AUC of 0.740 [SE = 0.046, 95% CI: 0.650–0.831] (Figure 6A). The validation data showed that the levels of let-7f-2*, miR-628-5p, miR-203, miR-200b and miR-141 were significantly different between stage I–II and stage III–IV CRC patients. The best logistic model, logit (p) = 9.181 + 0.186* miR-203, was established to distinguish between stage I–II and stage III–IV CRC patients. At the optimal cut-off, the predicated probability on the basis of plasma miR-203 had 74.7% sensitivity and 71.4% specificity, with an AUC of 0.757 [SE = 0.041, 95% CI: 0.676–0.838] (Figure 6B). According to the results from the validation cohort, plasma let-7f-2*, miR-628-5p, miR-96, miR-148a, miR-203, miR-200b, and miR-141 levels were significantly different between stage I–III and stage IV CRC patients. A logistic regression analysis demonstrated the best logistic model based on miR-141, logit (p) =13.888+0.395*miR-141. At the optimal cut-off, the model had 80.0% sensitivity and a 86.1% specificity for distinguishing between stage IV and stage I–III CRC patients, with an AUC of 0.851 [SE = 0.046, 95% CI: 0.762–0.940] (Figure 6C).

### Survival analyses for CRC patients

**Figure 7:**
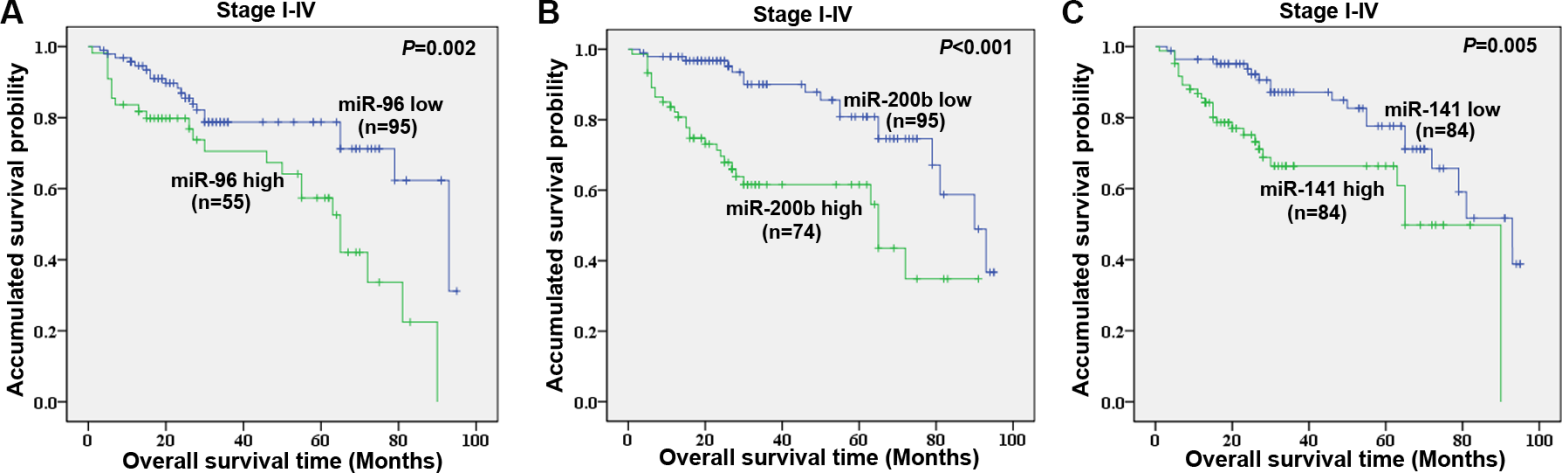
Univariate survival analyses of plasma miR-96 (A), miR-200b (B), and miR-141 (C) levels in stage I–IV CRC patients in the validation cohort using the Kaplan-Meier method and log-rank test

We performed univariate survival analysis for the 11 candidate miRNAs identified from screening data in the validation cohort. Plasma miR-96, miR-200b and miR-141 were demonstrated to be associated with overall survival in CRC patients using a univariate survival analysis (Figure 7A–7C). The patients with lower levels of plasma miR-96 (*P* = 0.002), miR-200b (*P* < 0.001), and miR-141 (*P* = 0.005) showed better survival rate than those with higher levels of the corresponding miRNA. The multiple survival analysis, including known clinical parameters, serum CEA, and the three plasma miRNAs, revealed that stage, serum CEA, plasma miR-200b (*P* = 0.008, risk ratio (RR) = 2.630), and miR-96 (*P* = 0.019, RR = 2.275) were independent factors for overall survival of CRC patients (Table 2).

**Table 2:** Multivariate analyses of overall survival in 187 CRC patients

Factor	*P* value	Risk ratio	95% CI
Stage (III–IV *vs* I–II)	0.001	4.746	1.917–11.751
CEA (> 5 μg/l *vs* ≤ 5 μg/l)	0.001	3.412	1.612–7.219
miR-200b (high *vs* low)	0.008	2.630	1.287–5.375
miR-96 (high *vs* low)	0.019	2.275	1.147–4.512
Sex (female *vs* male)	0.307	0.683	0.328–1.420
miR-141 (high *vs* low)	0.335	1.555	0.778–3.106
Chemotherapy (yes *vs* no)	0.396	0.684	0.284–1.646
Age (≥ 55 years *vs* < 55 years)	0.575	1.232	0.594–2.552

## DISCUSSION

In our two-tiered study, parts of the results from the discovery phase were clinically validated in an independent large-scale cohort. Our results suggest that the high throughput method of miRNA profiling in a sufficient number of plasma samples could identify candidate biomarkers. The logistic regression, ROC curve, and survival analyses identified the most significant miRNAs for potential clinical use, and the detailed analysis in healthy controls and CRC patients with each stage supplied candidates (miR-96, miR-203, miR-141 and miR-200b) for prognosis of CRC patients if clinical utility studies are completed successfully. The proposed functions and expression of the four candidate miRNAs in CRC tissue and circulation from literature [11, 18, 22, 25–49] were summarized in Table S7.

miR-96, a member of the miR-183 family, has been identified as a potential oncogene in several tumor types [26, 50–55]. Several studies showed that miR-96 was upregulated in CRC tissues compared to in normal mucosal tissue [25–31], and miR-96 in CRC tissue was found to be associated with liver metastasis [29]. These results in CRC tissue are consistent with our data about miR-96 in plasma. In the current study, plasma miR-96 exhibited significantly higher levels in all CRC patients than healthy controls. Plasma miR-96 levels in stage IV CRC patients were significantly higher than those in stage I–III CRC patients. Moreover, plasma miR-96 level was suggested to be an independent prognostic marker for CRC patients. Brunet Vega *et al.* examined 11 miRNAs in the sera of 30 stage III CRC patients and 26 healthy controls and found that the mean fold change in serum miR-96 levels was 2.267, although the difference was not significant [30]. We obtained similar results in our study. Plasma miR-96 levels in stage I, II and IV CRC patients were significantly higher than those in healthy controls, but there was only a trend that plasma miR-96 levels in stage III CRC patients were higher than those in controls. Although we do not know the reason why plasma miR-96 levels decreased from stage II to stage III, the difference of miR-96 levels between stage II and stage III was identified in the screening phase and validated in the validation cohort. Considering the lymph node metastasis cannot be identified accurately by iconography before surgery, plasma miR-96 level may potentially contributes to distinguishing stage II and stage III CRC, which will help oncologist choose suitable treatment for these CRC patients. Therefore, plasma miR-96 is a potentially useful clinical marker in CRC prognosis and treatment, especially in stage II and III CRC patients.

The biological role of miR-203 is heterogeneous. miR-203 has been reported to function as a tumor suppressor in head and neck squamous cell carcinomas, esophageal squamous cell carcinoma, and cervical and prostate cancer [56–58]; miR-203 was suggested to be an oncogene in pancreatic, kidney and ovarian cancers [59–61]. The role of miR-203 in CRC is also inconsistent [32, 34–39]. Bovell *et al*. found higher expression of miR-203 in CRC than in corresponding normal tissues, and high miR-203 expression was associated with poor survival in white patients with stage IV CRC and black patients with stage I and II CRC [34]. Four other studies also reported that miR-203 in CRC tissue was higher than normal tissue [35–38]. However, two studies showed that miR-203 had a decreased expression in CRC tissue [32, 39], and the miR-203 downregulation was correlated with tumor size and pT stage [39]. Wang *et al.* reported that serum miR-203 levels were lower in CRC patients than in healthy controls [22]. In our study, plasma miR-203 levels were significantly higher in all CRC patients than in controls, and they were higher in stage III–IV than stage I–II CRC patients. The discrepancy across the different studies might be partly due to the difference in sample origin (serum or plasma), patient numbers, technology platforms, and the endogenous controls for normalization. Furthermore, circulating miR-203 appears to be a highly dynamic miRNA that changes under different physiological conditions. Further studies are needed to evaluate the role of circulating miR-203 in CRC.

miR-141 and miR-200b belong to the miR-200 family that is known to be a regulator of epithelial-to-mesenchymal transition (EMT) [41, 48]. Although miR-141 was demonstrated to inhibit CRC cell migration and invasion *in vitro* [43], significantly upregulated miR-141 expression was observed in the plasma of metastatic CRC patients [11, 45]. Consistent with our previous study [11], the present study demonstrated that plasma miR-141 was a potential biomarker for metastatic CRC again. Recent studies have revealed complex functions associated with miR-200 family members [48]. The EMT inhibitor miR-200b was shown to stimulate tumor growth in TGFBR2-null CRC by targeting CDKN1B and negatively regulating p27/kip1 [46]. Toiyama et al. didn't find the significant difference of serum miR-200b levels between stage I and stage IV patients in their identification phase. However, they showed that serum miR-200c levels were significantly higher levels in stage IV than stage I–III CRC patients, and high serum miR-200c levels were positively correlated with lymph node metastasis, distant metastasis, and prognosis and were an independent prognostic marker for CRC [18]. In our current study, we observed that plasma miR-200b levels were higher in stage III–IV CRC, especially in metastatic CRC, and emerged as an independent prognostic marker for CRC patients.

We did not confirm most circulating miRNAs that were reported previously. One of the reasons may be that we performed many comparisons between different groups and chose relatively fewer miRNAs as candidates based on each comparison in the discovery phase, so that some miRNAs with relatively lower significance were not selected in our study. However, the inconsistency of the results from multiple studies on circulating miRNAs has been reported previously and clearly impedes their clinical usage [62, 63]. The lack of reproducibility could be attributed to factors such as sample size, sample preparation, screening method, and data normalization and analysis. Data normalization has been a troubling factor because no standard reference miRNA has been established [24]. U6 was demonstrated not to be suitable as endogenous control for the quantification of circulating microRNAs [64]. Although miR-16 was used as an internal control in some studies of miRNAs [9, 15], the circulating levels of miR-16 were demonstrated to be associated with hemolysis and bowel preparation [65]. In addition, circulating miR-16 was suggested to be a potential biomarker in gastric, breast and hepatocellular cancer [66–68], suggesting it is not a stable internal control for blood samples. Our results showed that plasma levels of miR-451 were more stable among healthy controls and CRC patients with each stage. miR-451 was recommended as an internal control in other study [69]. In addition, the validation results for selected candidate miRNAs using spiked-in cel-miR-39 or miR-451 as internal control were consistent in this study. The data suggest that miR-451 serves as a stable control miRNA for circulating miRNA analyses, although it must be further validated to establish a standardized protocol for clinical use.

Although the plasma levels of let-7f-2*, miR-628, miR-96, miR-148a, miR-203, miR-200b, miR-22 and miR-141 were significantly different between CRC patients and controls in our validation phase, detailed analyses of each stage showed that the difference between CRC patients and controls might result from the remarkable difference between advanced stage CRC and controls. Margue *et al*. also found that the levels of cell-free miRNAs only change significantly at later stages of melanoma progression [70]. Thus, development of clinically useful circulating markers for cancer detection should consider both early and late stages of cancers.

In conclusion, on the basis of a microRNAome screening of 50 individual samples and clinical validation in a relatively large sample of 284 cases, we propose four miRNA candidates as non-invasive biomarkers for CRC prognosis. We believe these biomarkers have the potential to be useful in clinical settings and prospective studies of clinical utility are warranted. Of note, the robustness of these potential biomarkers on clinical benefit needs to be evaluated in the future by using an independent CRC sample cohort.

## MATERIALS AND METHODS

### Patient characteristics and specimens

This study was approved by The University of Texas MD Anderson Cancer Center (Houston, Texas) institutional review board. Plasma samples from healthy individuals and CRC patients (stages I–IV) were obtained from TexGen between 2002 and 2010, a collaboration of Texas Medical Center institutions that provides biological samples as well as epidemiological and clinical data [11]. Written informed consent had been obtained from all patients for use of specimens and clinical data. Blood samples from cancer patients had been obtained before colorectal surgical resection. Patients who had undergone preoperative radiotherapy or chemotherapy were excluded. Tumors were staged according to the American Joint Committee on Cancer TNM staging system for colorectal cancer [71]. To select samples in healthy individuals, we used asymptomatic and apparently healthy volunteers with no history of cancer. All volunteers were confirmed to be healthy, with no malignancy, by physical examination. The serum CEA levels and clinical data (including gender, age, race and stage) were acquired from the TexGen clinical database that had been developed from clinical records. Follow-up data on all CRC patients were acquired from TexGen clinical database, and the survival time was calculated from the date of diagnosis to the date of death or last follow-up in October 2010. The median follow-up time was 28 months.

### RNA isolation from plasma

Small RNA was enriched from all plasma samples using the *mir*Vana PARIS RNA isolation kit (Ambion, Austin, TX). In brief, a 300 μL aliquot of plasma was thawed on ice and centrifuged at 14,000 rpm for 10 minutes to remove cells and cellular debris. Next, 250 μL of supernatant was lysed with an equal volume of 2x denaturing solution. For normalization of sample-to-sample variation during the RNA isolation procedures, 25 fmol of synthetic C. elegans miRNA (cel-miR-39, GE Dharmacon) was added to each denatured sample. Small RNAs were then enriched and purified following the manufacturer's protocol, with the exception that the enriched small RNAs were eluted in 20–30 μL of preheated nuclease-free water. DNase (Qiagen) treatment was used to remove any contaminating DNA. The RNA concentration was quantified using NanoDrop1000 (NanoDrop, Wilmington, DE) in all samples and ranged from 3 to 35 ng/μL.

### miRNA screening assay

Complementary DNA (cDNA) was generated for each RNA sample from the plasma of the discovery set using reverse transcription with Megaplex RT primers, human pool set v3.0 (Life Technologies, Foster City, CA), which consists of 754 stem-looped reverse transcription primers plus three TaqMan-designated internal controls. The cDNA underwent pre-amplification with Megaplex PreAmp Primers, human pool set v3.0 (Life Technologies), and was loaded onto the TaqMan array human microRNA A + B cards set v3.0 (Life Technologies). These cards were run using the Applied Biosystems 7900HT real-time PCR system, according to the manufacturer's instructions.

Although the TaqMan array provides three internal controls (U6, RNU44, and RNU48) that are commonly used to measure cellular miRNAs, there are no standard endogenous control miRNAs, especially for an analysis of circulating miRNAs [24]. We first preprocessed the miRNAs by filtering out those with the same raw cycle threshold (Ct) value in all samples in the whole cohort, corresponding to a standard deviation of zero. We next chose a normalization reference from the preprocessed miRNAs that was based on the following three criteria [64, 72]: (1) Small expression variation, in terms of standard deviation across the whole discovery cohort; (2) High RNA yield or absorbance in terms of the raw Ct values; and (3) No statistically significant difference in miRNA expression among samples in different categories (i.e., healthy controls *vs* CRC patients, or among stage I, II, III and IV CRC patients). The normalization was performed by subtracting the raw Ct values of the reference miRNA from the Ct values of all the other miRNAs and was expressed as ΔCt. ΔΔCt was then calculated by subtracting the average ΔCt of the control from ΔCt of all samples. The relative expression levels of miRNAs were then calculated utilizing the -ΔΔCt method. The analysis was performed by using the Matlab software. The raw and processed data for Card A and Card B were shown in Table S2.

To determine whether candidate miRNAs could serve as effective markers, we performed several pairwise comparisons (control *vs* CRC, control *vs* stages I–II, control *vs* stage III–IV, stage I–II *vs* stage III–IV, control *vs* stage IV, stage I–III *vs* stage IV, and stage II *vs* stage III). miRNAs that were significantly differentially expressed in these comparisons were chosen as potential candidates for further investigation.

### miRNA clinical validation study

Eleven miRNAs selected from the discovery phase were analyzed using real time RT-PCR assays as described previously [11]. Expression levels of miRNAs were quantified in duplicate and spiked-in cel-miR-39 was used as normalizers for plasma miRNA quantification. The mean cycle threshold of the replicated measurement of each miRNA was included in the analysis. The software defaults of the 7900 Sequence Detection System 2.3 (Applied Biosystems) were used to compute the relative change in RNA expression with the ΔCt method and 95% confidence intervals.

### Statistical analysis

Standard statistical tests including Student's *t*-test and Mann-Whitney test were used to assess difference in miRNA levels between different groups. In the validation cohort, receiver operating characteristic (ROC) curves [73] were generated to assess the diagnostic accuracy of each parameter, and the sensitivity and specificity of the optimum cut-off point were defined as those values that maximized the area under the ROC curve (AUC). Multivariate logistic regression analysis was performed to find the best miRNA panel for CRC detection or stage [9]. The AUC was used as an accuracy index for evaluating the diagnostic performance of the selected microRNA panels. The associations between overall survival and plasma miRNAs were analyzed using the Kaplan-Meier method and the log-rank test [74]. For each miRNA, the patients were divided into two groups (low and high levels) based on the mean of the relative expression levels of the miRNA in plasma. A Cox proportional-hazards regression analysis was used to evaluate the independent prognostic factors. Statistical analysis was performed using the software packages such as Matlab (MathWorks, Inc., Natick, Massachusetts), SPSS version 16.0 (WPSS, Ltd., Surrey, United Kingdom) and GraphPad Prism 5.0 (GraphPad Software, Inc., California). All statistical tests were two-sided, and a *P* value of less than 0.05 was considered significant.

## SUPPLEMENTARY MATERIALS FIGURES AND TABLES






