# Supplementary material for: Examining plasma microRNA markers for colorectal cancer at different stages
Source: Oncotarget. 2016 Feb 4;7(10):11434–49. doi: 10.18632/oncotarget.7196 (PMC4905484; doi:10.18632/oncotarget.7196)
Supplement: Supplementary file 1 [file oncotarget-07-11434-s001.pdf]

Examining plasma microRNA markers for colorectal cancer at different stages

Supplementary Materials

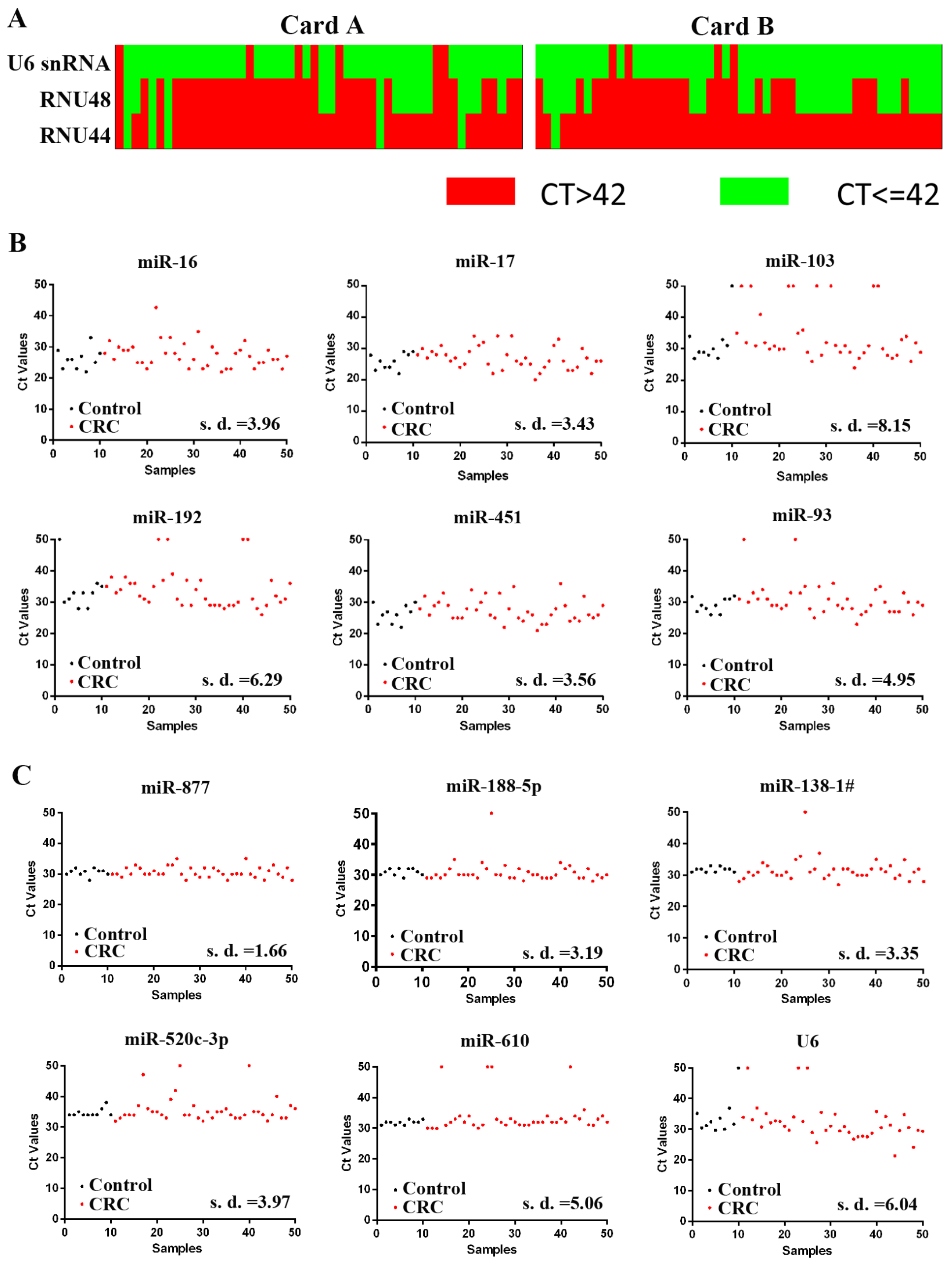

Supplementary Figure S1: Selection of normalization references for card A and card B. (**A**) Relative cycle threshold (Ct) values of the three endogenous miRNAs in cards A and B. The red color denotes a Ct value of > 42, and the green color indicates a Ct value of ≤ 42. (**B**) Distribution of the raw Ct values of the six miRNAs with the least standard deviation across the entire cohort in card A. miR-451 was chosen for the card A normalization reference. (**C**) Distribution of the raw Ct values of the six miRNAs with the least standard deviation across the entire cohort in card B. miR-877 was chosen for the card B normalization reference. The black dots denote healthy controls and the red dots denote CRC samples. s.d. indicates standard deviation.

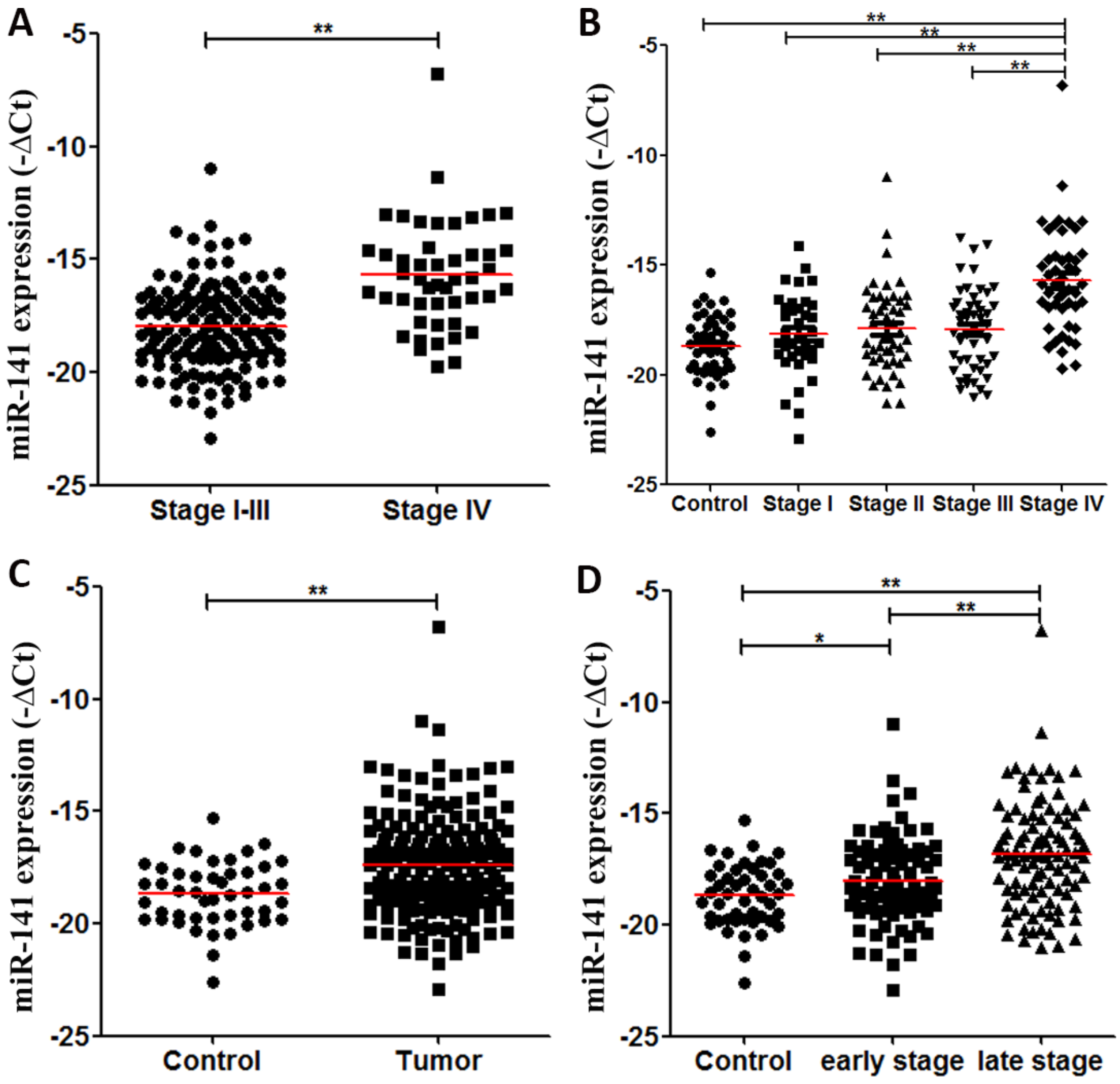

Supplementary Figure S2: Levels of miR-141 in plasma from 187 CRC patients and 47 healthy controls tested by real-time RT-PCR and normalized with cel-miR-39 as the control. (**A**) Levels of plasma miR-141 in stage IV and stage I–III CRC patients. (**B**) A detailed analysis of plasma miR-141 levels in healthy controls and stage I–IV CRC patients. (**C**) Plasma miR-141 levels in all CRC patients and healthy controls. (**D**) Plasma miR-141 levels in controls and stage I–II and stage III–IV CRC patients. *$P < 0.05$; **$P < 0.01$. The *p*-values were shown in Table S5.

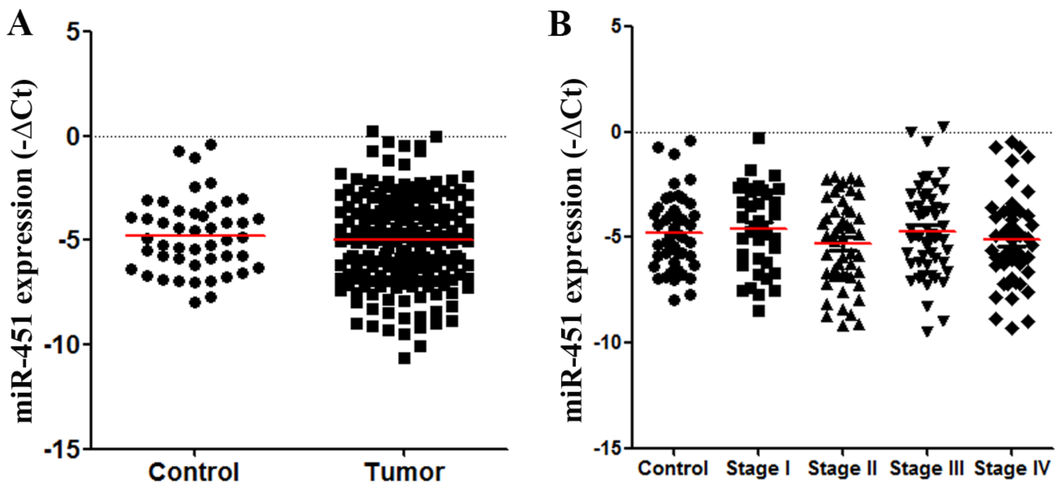

Supplementary Figure S3: Levels of miR-451 in plasma from 187 CRC patients and 47 healthy controls tested by real-time RT-PCR and normalized with cel-miR-39 as the control in the validation set. (**A**) Levels of plasma miR-451 in all CRC patients and healthy controls ($P = 0.479$). (**B**) A detailed analysis of plasma miR-451 levels in healthy controls and stage I–IV CRC patients. There was no significant difference between each group (healthy controls vs stage I, $P = 0.647$; controls vs stage II, $P = 0.115$; controls vs stage III, $P = 0.930$; controls vs stage IV, $P = 0.284$; stage I vs stage II, $P = 0.069$; stage I vs stage III, $P = 0.736$; stage I vs stage IV, $P = 0.173$; stage II vs stage III, $P = 0.121$; stage II vs stage IV, $P = 0.660$; stage III vs stage IV, $P = 0.279$).

Supplementary Table S1: Summary of 16 articles about circulating miRNAs as potential diagnostic or prognostic biomarkers for CRC patients

Reference	Identification phase					Validation phase				Conclusion
	Method	# testing miRNAs	Normalizer	#samples	Differentiated miRNAs	Method	miRNAs for validation	Normalizer	#samples	
Ng et al. [8]	real-time PCR-based miRNA profiling	95	U6	5 CRCs and 5 HCs	5 miRNAs ↑ (miR-17-3p, miR-135b, miR-222, miR-92 and miR-95) in CRC patients	qRT-PCR	5 miRNAs in selection and validation;<br>2 miRNAs (miR-17-3p and miR-92) in an independent validation	RNU6B	Selection and validation: 25 from CRC and 20 from HC;<br>Independent validation: 90 from CRC, 20 from GC, 20 from IBD and 50 from HC	Plasma miR-92 was a potential biomarker for CRC detection.
Huang et al. [9]	qRT-PCR	12	miR-16	20 CRCs and 20 HCs	2 miRNAs ↑ (miR-29a and 92a), 1 miRNA ↓ (miR-25) in CRC patients	qRT-PCR	miR-29a and 92a	miR-16	80 from CRC, 37 from AA and 39 from HC	Plasma miR-29a and miR-92a were potential biomarkers for early detection of CRC.
Pu et al. [10]	qRT-PCR	3	no control	103 CRCs and 37 HCs	miR-221↑	/	/	/	/	Plasma miR-221 can be used as a potential marker for diagnosis and prognosis of CRC.
Cheng et al. [11]	qRT-PCR	3	cel-miR-39	74 CRCs and 28 HCs	miR-141↑ in stage IV CRC	qRT-PCR	miR-141	cel-miR-39	108 CRCs and 48 HCs	Plasma miR-141 is a potential biomarker for metastatic CRC and predicts poor prognosis.
Ahmed, et al. [12]	Affymetrix Gene Chip Micro 3.0 Array	19,724 miRNAs in 153 species	using a variation stabilization transformation method	15 CRCs and 5 HCs,	176 miRNAs (122 ↑ and 54↓) in CRC plasma and tissue samples	qPCR	15 selected miRNAs (9↑ and 6↓)	18S rRNA	The same samples in identification phase	9 miRNAs (miR-7, -17-3p, -20a, -21, -92a, -96, -183, -196a and -214) and 6 miRNAs‾ (miR-124, -127-5p, -138, -143, -146a and -222) in CRC plasma and tissue
Kanaan, et al. [13]	qRT-PCR	6	U6	30 CRCs and 30 HCs	1 miRNA (miR-21)↑ in CRC patients	qPCR	miR-21	U6	20 CRCs and 20s HCs	Plasma miR-21was a potential diagnostic marker of CRC.
Wang et al. [14]	miRCURY LNATM Universal RT microRNA PCR system (Exiqon)	742	cel-miR-39 and miR-16	one pool of 10 CRCs (5 stage II and 5 stage III) and the other pool of 10 HCs	86 miRNAs differentially expressed	qPCR	17 miRNAs (9 ↑ and 13↓ miRNAs)	cel-miR-39	90 CRCs and 58 HCs	Plasma miR-601 and miR-760 can potentially serve as novel biomarkers for the early detection of CRC.
Giraldez, et al. [15]	SAM-Bead Array platform (Illumina)	743	using the Lumi bioconductor package	21 CRCs, 20 AAs and 20 HCs	93 and 125 miRNAs deregulated in CRC and in AA, respectively, sharing 24 miRNAs↑	qRT-PCR	12 miRNAs from the top 50 deregulated miRNAs in CRC and/or AAs, and 2 additional miRNAs (miR19a and miR20a)	miR-16	42 CRCs and 53 HCs	6 miRNAs (miR-18a, -19a, -19b, -15b, -29a and -335) in CRC, compared with controls.<br>miR-18a overexpressed in AA patients in comparison with controls.

Luo et al. [16]	TaqMan MicroRNA Array (Applied Biosystems)	667	miR-16	5 pools of CRCs (n = 50) and 5 pools of controls (n = 50)	5 miRNAs↑ (miR-29a, -106b, -133a, -342-3p, -532-3p)	qPCR	The above 5 miRNAs and another 7 miRNAs previously reported (miR-18a, -20a, -21, -92a, -143, -145, -181b)	miR-16	80 CRCs, 50 AAs and 194 HCs	The panel of miR-18a, miR-20a, miR-21, miR-29a, miR-92a, miR-106b, miR-133a, miR-143 and miR-145 could be of potential use in the early detection of CRC.
Yong et al. [17]	GeneChip miRNA 2.0 Array (Affymetrix)	1,105	Median intensity values for each miRNA from the same replicates were subjected to quantile normalization to normalize the data across different arrays	42 CRCs in 8 pools and 18 HCs in 6 pools	15 miRNAs↑ and 9 miRNAs↓	stem-loop RT-PCR	7 miRNAs (miR-150, miR-193a-3, miR-23a, miR-23b, miR-338-5p, miR-342-3p and miR-483-3p) concurrently deregulated in blood and tissue in identification phase	RNU48	70 CRCs and 32 healthy controls	The triple miRNA classifier of miR-193a-3p, miR-23a and miR-338-5p could be a potential biomarker in the detection of CRC.
Toiyama et al. [18]	qRT-PCR	4 miRNAs (miR-200b, -200c, -141, and -429)	cel-miR-39	12 stage I and 12 stage IV CRC patients	miR-200c↑ in stage IV CRC patients compared to stage I	qRT-PCR	miR-200c	cel-miR-39	182 CRCs and 24 controls	Serum miR-200c was an independent predictor for lymph node metastasis, tumor recurrence and an independent prognostic marker for CRC.
Kanaan et al. [19]	TaqMan low-density array card (Life Technologies)	380	Not mention	9 AAs, 20 stage III and IV CRCs and 12 HCs	10 miRNAs most significantly dysregulated in CRC patients (miR-192, -431, -15b, -139-3p, -21, -331, -423-5p, -339-3p, -142-3p, -25)	qRT-PCR	10 miRNAs from identification phase	U6	16 AAs, 15 stage I-II, 15 stage III, 15 stage IV, and 26 HCs	The panel of miR-431 and miR-139-3p for CRC diagnosis (vs controls), the panel of miR-331, miR-15b and miR-21 for stage IV CRC (vs controls), and the panel of miR-331, miR-15b, miR-21, miR-142-3p and miR-339-3p for adenomas (vs CRC)
Kjersem, et al. [20]	miRCURY LNA Universal RT microRNA PCR system (Exiqon)	742	the average of 81 miRNAs detected in 44 of the 48 samples	48 CRCs (before and after treatment in 12 responders and 12 non-responders)	22 miRNAs were associated with response to treatment.	qPCR	32 miRNAs (22 miRNAs from identification phase and 10 miRNAs which will be used as endogenous controls)	the average of three miRNAs (miR-126,miR-15b, and miR-24)	150 samples (90 respondors and 60 non-respondors)	In metastatic CRC patients with 5-FU and oxaliplatin-based first-line chemotherapy, miR-106a, miR-130b, and miR-484 were associated with lack of response, whereas miR-27b, miR-148a, and miR-326 were associated with reduced PFS.

Shivapurkar et al. [21]	qRT-PCR	16	U6	10 CRCs (5 with recurrence and 5 without recurrence	Neither reached statistical significance.					
	SYBR Green qPCR based genome-wide miR expression arrays	760	/	1 from recurrence-free group and 1 from recurrence group	showed the top 70 miRNAs differentially expressed	qRT-PCR	6	U6	30 early-stage CRCs (15 with recurrence and 15 recurrence-free)	The miRNA cluster (miR-15a, -148a, -320a, -451, -103 and -596) can be used to predict the risk of disease recurrence of early stage CRC.
Wang et al. [22]	qRT-PCR	10	miR-16	30 CRCs and 30 HCs	Six miRNAs (miR-21, -31, -92a, -181b, -203, and let-7g) differentially expressed	qRT-PCR	6	miR-16	83 CRCs and 59 HCs	The identified six-miRNA signature can be used as a noninvasive biomarker for the diagnosis of CRC.
Du et al. [23]	meta-analysis	/	/	/	miR-21<br>miR-92a	qRT-PCR	2	cel-miR-39	49 CRCs and 49 HCs	Plasma miR-21, but not miR-92a, could be a reliable and non-invasive biomarker for CRC diagnosis.

Supplementary Table S2: The raw data and processed data of all miRNAs in the discovery phase

Supplementary Table S3: Statistical significance between healthy controls and CRC patients in the discovery cohort (*P*-values)

Supplementary Table S4: The pairwise comparisons of plasma candidate miRNAs in discovery phase (*P*-value§)

microRNAs	P *vs.* C	$S_{I–II}$ *vs.* C	$S_{III–IV}$ *vs.* C	$S_{III–IV}$ *vs.* $S_{I–II}$	S_{IV} *vs.* $S_{I–III}$	S_{III} *vs.* S_{II}
**let-7f-2***	H (0.008)	NS (0.060)	H (0.013)	NS (0.453)	NS (0.983)	NS (0.952)
**miR-15b***	H (< 0.001)	H (0.021)	H (0.008)	NS (0.653)	NS (0.410)	NS (0.581)
**miR-526b**	H (< 0.001)	H (< 0.001)	H (< 0.001)	NS (0.714)	NS (0.998)	H (0.158)
**miR-628-5p**	H (< 0.001)	H (0.008)	H (0.008)	NS (0.846)	NS (0.751)	NS (0.790)
**miR-486-3p**	H (0.017)	NS (0.090)	H (0.010)	NS (0.423)	NS (0.559)	NS (0.743)
**miR-801**	L (< 0.001)	L (0.001)	L (0.006)	NS (0.509)	NS (0.675)	NS (0.659)
**miR-376c**	L (< 0.001)	L (< 0.001)	L (0.030)	NS (0.517)	NS (0.249)	NS (0.783)
**miR-96**	NS (0.174)	H (0.031)	NS (0.754)	L (0.048)	NS (0.817)	L (0.024)
**miR-30a-5p**	NS (0.254)	L (0.037)	NS (0.749)	H (0.010)	NS (0.312)	NS (0.086)
**miR-766**	NS (0.103)	L (0.027)	NS (0.977)	H (0.020)	NS (0.124)	NS (0.112)
**mir-197**	NS (0.374)	L (0.039)	NS (0.430)	H (0.001)	H (0.001)	NS (0.108)
**miR-148a**	L (0.003)	L (< 0.001)	NS (0.059)	H (0.022)	H (0.024)	NS (0.180)
**miR-130b**	L (0.011)	L (0.002)	NS (0.213)	H (0.043)	H (< 0.001)	NS (0.917)
**miR-22**	L (0.019)	NS (0.170)	L (0.003)	L (0.018)	NS (0.578)	NS (0.070)
**miR-203**	NS (0.130)	NS (0.562)	H (0.035)	H (0.044)	H (0.012)	NS (0.813)
**miR-200b**	H (0.022)	NS (0.177)	H (0.005)	H (0.028)	NS (0.062)	NS (0.616)
**miR-31**	NS (0.241)	NS (0.064)	NS (0.792)	NS (0.100)	H (< 0.001)	NS (0.282)
**miR-141**	NS (0.560)	NS (0.556)	NS (0.663)	NS (0.918)	H (0.056)	NS (0.167)
**miR-191**	NS (0.649)	NS (0.994)	NS (0.379)	NS (0.122)	H (0.006)	NS (0.975)
**miR-155**	NS (0.860)	NS (0.674)	NS (0.383)	NS (0.188)	H (0.002)	NS (0.913)
**miR-126**	NS (0.295)	NS (0.449)	NS (0.201)	NS (0.263)	H (0.023)	NS (0.697)
**miR-519b-3p**	NS (0.676)	NS (0.853)	NS (0.339)	NS (0.102)	L (0.003)	NS (0.946)

P, patients with CRC; C, healthy control; S_{I-II}, stage I-II CRC patient; S_{III-IV}, stage III-IV CRC patient; S_{I-III}, stage I-III CRC patient; S_{IV}, stage IV CRC patient; H, higher; L, lower. §Student's *t*-test without assumption of equal variances.

Supplementary Table S5: Comparisons of plasma levels of 11 candidate microRNAs in the validation phase using cel—miR-39 for normalization (*P*-value)

microRNAs	P *vs.* C	S_I vs. C	S_{II} *vs.* C	S_{III} *vs.* C	S_{IV} *vs.* C	$S_{I–II}$ *vs.* C	$S_{III–I}$V *vs.* C	$S_{III–IV}$ *vs.* $S_{I–II}$	S_{IV} *vs.* $S_{I–III}$	S_{III} *vs.* S_{II}
**let-7f-2***	H (0.019)	NS (0.302)	NS (0.402)	NS (0.088)	H (0.001)	NS (0.268)	H (0.003)	H (0.026)	H (0.010)	NS (0.446)
**miR-628-5p**	H (0.037)	NS (0.331)	NS (0.053)	NS (0.057)	H (< 0.001)	NS (0.495)	H (0.003)	H (0.006)	H (0.010)	NS (0.937)
**miR-486-3p**	NS (0.604)	NS (0.509)	NS (0.762)	NS (0.738)	NS (0.242)	NS (0.922)	NS (0.397)	NS (0.402)	NS (0.264)	NS (0.558)
**miR-526b**	NS (0.616)	NS (0.205)	NS (0.477)	NS (0.362)	H (0.014)	NS (0.827)	NS (0.524)	NS (0.680)	H (0.008)	NS (0.917)
**miR-15b***	NS (0.124)	NS (0.289)	NS (0.195)	NS (0.637)	H (0.032)	NS (0.167)	NS (0.143)	NS (0.748)	NS (0.117)	NS (0.500)
**miR-96**	H (0.003)	H (0.019)	H (0.009)	NS (0.461)	H (< 0.001)	H (0.003)	H (0.007)	NS (0.662)	H (0.010)	L (0.043)
**miR-148a**	H (0.003)	NS (0.355)	H (0.041)	H (0.037)	H (< 0.001)	NS (0.064)	H (0.001)	NS (0.052)	H (0.006)	NS (0.960)
**miR-203**	H (0.003)	NS (0.133)	NS (0.237)	NS (0.165)	H (< 0.001)	NS (0.107)	H (< 0.001)	H (0.006)	H (< 0.001)	NS (0.881)
**miR-200b**	H (0.012)	NS (0.522)	NS (0.489)	H (0.029)	H (< 0.001)	NS (0.867)	H (< 0.001)	H (< 0.001)	H (< 0.001)	NS (0.244)
**miR-22**	H (0.019)	NS (0.199)	NS (0.119)	H (0.027)	H (0.016)	NS (0.098)	H (0.009)	NS (0.264)	NS (0.490)	NS (0.563)
**miR-141**	H (< 0.001)	NS (0.132)	H (0.027)	H (0.031)	H (< 0.001)	H (0.034)	H (< 0.001)	H (< 0.001)	H (< 0.001)	NS (0.903)

P, patients with CRC; C, healthy control; S_I, stage I CRC patient; S_{II}, stage II CRC patient; S_{III}, stage III CRC patient; S_{IV}, stage IV CRC patient; $S_{I–II}$, stage I–II CRC patient; $S_{III–IV}$, stage III–IV CRC patient; $S_{I–III}$, stage I-III CRC patient; H, higher; L, lower; NS, no significance.

Supplementary Table S6: Comparisons of plasma levels of 11 candidate microRNAs in the validation phase using miR-451 for normalization (*P*-value)

microRNAs	P *vs.* C	S_I *vs.* C	S_{II} *vs.* C	S_{III} *vs.* C	S_{IV} *vs.* C	$S_{I–II}$ *vs.* C	$S_{III–IV}$ *vs.* C	$S_{III–IV}$ *vs.* $S_{I–II}$	S_{IV} *vs.* $S_{I–III}$	S_{III} *vs.* S_{II}
**let-7f-2***	H(0.025)	NS (0.580)	NS (0.082)	NS (0.246)	H (0.001)	NS (0.175)	H (0.011)	NS (0.146)	H (0.013)	NS (0.601)
**miR-628-5p**	H (0.038)	NS (0.209)	H (0.013)	NS (0.128)	H (< 0.001)	NS (0.411)	H (0.005)	H (0.014)	H (0.002)	NS (0.381)
**miR-486-3p**	NS (0.148)	NS (0.835)	NS (0.071)	NS (0.732)	H (0.031)	NS (0.211)	NS (0.152)	NS (0.839)	NS (0.100)	NS (0.143)
**miR-526b**	NS (0.441)	NS (0.349)	NS (0.713)	NS (0.510)	H (0.006)	NS (0.811)	NS (0.317)	NS (0.401)	H (0.002)	NS (0.828)
**miR-15b***	NS (0.075)	NS (0.381)	NS (0.064)	NS (0.698)	H (0.015)	NS (0.102)	NS (0.098)	NS (0.755)	H (0.046)	NS (0.179)
**miR-96**	H (0.015)	NS (0.098)	H (0.022)	NS (0.871)	H (< 0.001)	H (0.018)	H (0.033)	NS (0.927)	H (0.009)	L (0.036)
**miR-148a**	H (0.003)	NS (0.461)	H (0.024)	H (0.046)	H (< 0.001)	NS (0.059)	H (< 0.001)	NS (0.058)	H (0.008)	NS (0.765)
**miR-203**	H (0.015)	NS (0.302)	NS (0.277)	NS (0.307)	H (< 0.001)	NS (0.214)	H (0.002)	H (0.024)	H (< 0.001)	NS (0.870)
**miR-200b**	H (0.011)	NS (0.411)	NS (0.083)	NS (0.081)	H (< 0.001)	NS (0.549)	H (< 0.001)	H (< 0.001)	H (< 0.001)	NS (0.988)
**miR-22**	H (0.007)	NS (0.508)	H (0.004)	NS (0.075)	H (0.003)	H (0.030)	H (0.007)	NS (0.262)	NS (0.085)	NS (0.497)
**miR-141**	H (0.001)	NS (0.510)	H (0.003)	NS (0.163)	H (< 0.001)	H (0.025)	H (< 0.001)	H (0.023)	H (< 0.001)	NS (0.126)

P, patients with CRC; C, healthy control; S_I, stage I CRC patient; S_{II}, stage II CRC patient; S_{III}, stage III CRC patient; S_{IV}, stage IV CRC patient; $S_{I–II}$, stage I–II CRC patient; $S_{III–IV}$, stage III–IV CRC patient; $S_{I–III}$, stage I–III CRC patient; H, higher; L, lower; NS, no significance.

Supplementary Table S7: The proposed functions and expression of the four candidate miRNAs in CRC tissue and circulation

miRNA	Function in CRC	Expression in tissue			Plasma/serum levels		
		CRC vs normal	Stage	Prognosis	CRC vs control	Stage	Prognosis
**miR-96**	Promotes cell proliferation through inhibiting TP53INP1, FOXO1 and FOXO3a [25]	Up-regulated [25–31]	Higher expression in pMMR stage IV than stage II CRC [27]<br>High expression correlated with liver metastasis [29]	/	/	Compared with the levels in 26 healthy controls, the mean fold change of serum miR-96 levels in 30 stage III CRC patients was 2.267, but the difference was not significant [30].	/
**MiR-203**	Reduces cell proliferation through inhibiting Hakai expression [32]<br>Functions as a stemness-inhibitor in CRC cells [33]	Up-regulated [34–38]<br>Down-regulated [32, 39]	Low expression positively correlated with tumor size and pT stage [39]	High expression associated with poor survival in the Maryland test cohort, but not associated with survival in Hong Kong validation cohort [36]<br>High expression associated with poor survival of whites with stage IV CRC, and associated with poor survival of with stages I and II CRC [34]	Down-regulated [22]	/	/
**miR-141**	Promotes cell growth, cell cycle progression and invasion partly through targeting DLC1 [40]<br>Inhibits EMT through targeting TGBF2 [41]<br>Suppresses expression of stem cell factors [42]<br>Reduces SIP1 and inhibits cell migration and invasion [43]	Overexpressed [30, 40]<br>No significant difference [11]	No significant difference between Stage IV and stage I–II CRC [11]	High levels associated with longer overall and DFS in the CRC patients treated with fluoropyrimidines, but not an independent prognostic marker [44]	/	High levels associated with stage IV CRC [11,45]<br>No significant differences in serum levels between stage I and stage IV CRC patients [18]	High plasma levels associated with poor prognosis [11]
**miR-200b**	Stimulates cell proliferation and cell cycle progression by negatively regulating CDKN1B and RND3 [46]<br>Promotes proliferation via targeting RECK [47]<br>Involved in EMT and regulated by Ascl2 [48]	Up-regulated [46]	miR-200b-3p had decreased expression with more advanced tumors [49]	/	/	No significant differences in serum levels between stage I and stage IV CRC patients [18]	/
